# Genome‐Wide Association Studies of Cognitive Progression in Alzheimer’s Disease

**DOI:** 10.1002/alz.092503

**Published:** 2025-01-03

**Authors:** Ruyu Shi, Kang‐Hsien Fan, Oscar L. Lopez, Eleanor Feingold, Menghan Liu, Priyanka Gorijala, Carlos Cruchaga, M. Ilyas Kamboh

**Affiliations:** ^1^ School of Public Health, University of Pittsburgh, Pittsburgh, PA USA; ^2^ University of Pittsburgh Alzheimer’s Disease Research Center, Pittsburgh, PA USA; ^3^ Department of Psychiatry, Washington University School of Medicine, St. Louis, MO USA; ^4^ Washington University School of Medicine, Saint Louis, MO USA; ^5^ Hope Center for Neurological Disorders, Washington University School of Medicine, St. Louis, MO USA

## Abstract

**Background:**

Cognitive decline represents a significant and gradual clinical manifestation in individuals affected by Alzheimer’s disease (AD). Currently, there is a lack of effective treatments to delay its progression. Quantitative genome‐wide association studies (GWAS) have yielded limited insights into progression traits. The primary objective of this study was to identify underlying genetic variants in AD patients using longitudinal Mini‐Mental State Examination (MMSE) measures.

**Method:**

A total of 1,089 AD patients, including 410 with available whole genome‐sequencing (WGS) data from the University of Pittsburgh Alzheimer’s Disease Research Center (ADRC), and 1,117 AD patients from the Washington University in St. Louis were recruited and genotyped. GWAS were executed on four MMSE‐based cognitive decline phenotypes. Subsequently, a meta‐analysis was performed, followed by a functional exploratory analysis of identified top variants.

**Result:**

Distinct genetic variants were associated with each progression phenotype. Notably, a genome‐wide significant association was identified on chromosome 4 near the *TAPT1* (top SNP = rs79296051; MAF = 0.017; *p* = 3.47E‐08; z = ‐5.52) gene, linked explicitly to the annual MMSE percentage changes. Additionally, several suggestive associations (*p* < 1E‐05) were observed in the other decline phenotypes.

**Conclusion:**

We identified one genome‐wide significant association and multiple suggestive associations with MMSE‐based cognitive progression in AD patients. Replications of these results in independent larger cohorts are needed.

